# Identification of sulforaphane regulatory network in hepatocytes by microarray data analysis based on GEO database

**DOI:** 10.1042/BSR20194464

**Published:** 2021-02-10

**Authors:** Lei Gao, Jinshen Wang, Yuhua Zhao, Junhua Liu, Da Cai, Xiao Zhang, Yutao Wang, Shuqiu Zhang

**Affiliations:** 1 Institute of Quality Standard and Testing Technology for Agro-Products, Shandong Academy of Agricultural Sciences, Jinan, P.R. China; 2 Shandong Provincial Key Laboratory of Test Technology on Food Quality and Safety, Jinan, P.R. China; 3 Department of Gastrointestinal Surgery, Shandong Provincial Hospital Affiliated to Shandong First Medical University, Jinan, Shangdong Province, P.R. China

**Keywords:** bioinformatics analysis, hepatocyte, HSP90AA1, Sulforaphane

## Abstract

For the past several years, more and more attention has been paid to the exploration of traditional medicinal plants. Further studies have shown that more dietary consumption of cruciferous vegetables can prevent the occurrence of tumor, indicating the potential applications in the chemoprevention of cancer. Sulforaphane (SFN) has been identified by the National Cancer Institute as a candidate for chemopreventive research; it is one of several compounds selected by the National Cancer Institute’s Rapid Access to Preventive Intervention Development Program and is currently in use. In the present study, based on the data of Gene Expression Omnibus database (GEO), the gene expression profile of hepatocytes that were treated with SFN was analyzed. The ANOVA and Limma packets in R were used to analyze the differentially expressed genes (DEGs). On this basis, gene ontology (GO) function and Kyoto Encyclopedia of Genes and Genomes (KEGG) signaling pathway enrichment were further analyzed. The core gene HSP90-α (cytosolic), class A member 1 (HSP90AA1) was screened by protein–protein interaction (PPI) network established by STRING and Cytoscape software for further study. Finally, miRNAs targeted HSP90AA1 were predicted by miRanda. All in all, based on the data of GSE20479 chip, the molecular mechanism of SFN on hepatocytes was studied by a series of bioinformatics analysis methods, and it indicated that SFN might effect on the hepatocyte by regulating HSP90AA1.

## Introduction

For the past several years, more and more attention has been paid to the exploration of traditional medicinal plants in order to discover novel lead compounds on account of their plentiful complexity of secondary metabolites [1]. Extensive researches in laboratory animals and limited human epidemiological data indicate that various plant-derived compounds (phytochemicals) can reduce the risk of certain types of cancer [2]. Further studies have shown that more dietary consumption of cruciferous vegetables can prevent the occurrence of tumor, indicating the potential applications in the chemoprevention of cancer [3]. The naturally occurring isothiocyanate sulforaphane (SFN; 4-methylsulfinylbutyl isothiocyanate) from cruciferous vegetables, which is present in the form of glucosinolate glucoraphanin is the most studied [4]. When the cells are damaged, glucoraphanin (a type of glucosinolates (GLSs)), the precursor of SFN, is hydrolyzed by the enzyme myrosinase released by the plant myroain cells into SFN [5]. Furthermore, metabolism of SFN *in vivo* produces SFN-cysteine (SFN-Cys) and SFN-N-actyl-cysteine (SFN-NAC), which retain longer in circulation, and were more plentiful in lung and plasma than SFN [6]. SFN-Cys inhibits migration and invasion by regulating invasion-related proteins in a variety of cancer cells. Furthermore, SFN-NAC induces apoptosis by inhibiting lysozyme formation in non-small cell lung cancer (NSCLC) cells mediated by microtubule destruction. Because cell proliferation and death influence cell motility, SFN-Cys or SFN-NAC may inhibit migration and invasion through regulating Claudins or microtubule-mediated autophagy [7].

SFN is an effective inducer of antioxidant and phase-2 detoxifying enzymes. In addition to its antioxidant and protective effects, SFN is known to have protective effects in many pathological models and has been used in preclinical studies [8]. Furthermore, the cytoprotective effect of SFN is mainly achieved through the nuclear factor-erythroid 2-related factor 2 (Nrf2) dependent mechanism. Nrf2 induced by SFN has been clearly demonstrated to promote the integrity of the blood–brain barrier (BBB) and provide neuroprotection against ischemic injury [9]. However, the combined use of SFN and NAC reduced oxidative stress in brain and blood during epileptic process in the acquired epilepsy rat model induced by status epilepticus, and prominently improved the pathological results by blocking the onset of spontaneous epilepsy, reducing cell loss and rescuing the complications [10]. SFN decreases the activation of p63-iRHOM2 pathway in palmoplantar keratosis and squamous esophageal cancer syndrome (TOC) keratinocytes, leads to the decrease in oxidative stress, inflammation, proliferation and stress response K16, and increases apoptosis [11]. In addition, there is growing evidence that SFN can inhibit the growth of various types of cancer from different organs, which has aroused interest in the use of SFN in anti-cancer therapy [12]. The combination of SFN and quercetin has anti-migration effect on melanoma [13].

Chemical prevention of SFN is a hotspot of current research. SFN has been identified by the National Cancer Institute as a candidate for chemopreventive research; it is one of several compounds selected by the National Cancer Institute’s Rapid Access to Preventive Intervention Development Program and is currently in use. In several preclinical and clinical trials [14]. In addition, the website (www.clinicaltrials.gov) lists at least 20 registered human trials that are investigating the effectiveness of SFN or broccoli preparations in the treatment of cancer, viral infections and chronic obstructive pulmonary disease [15]. Ge et al*.* have shown that SFN can inhibit the activity of gastric cancer stem cells (CSCs) by inhibiting the activation of acoustic Hh pathway, which may become a promising intervention for gastric cancer [16].

Over the past decade, a large number of gene mutations and tissue gene expression profiles (‘disease characteristics’) have been associated with complex polygenic diseases. Previous studies have linked disease characteristics to drug characteristics using gene enrichment analysis, and identified candidate drugs for cancer, neurological and gastrointestinal diseases based on subsequent studies in cell lines and animal models [17]. In this study, we will analyze gene expression profiles of hepatocytes treated with SNF based on data from GEO public database, and preliminarily analyze the role of SFN in hepatocytes and its potential molecular mechanism by means of bioinformatics.

## Materials and methods

### Microarray data

GSE20479 based on the platforms—GPL1313 was obtained from GEO public database (https://www.ncbi.nlm.nih.gov/geo/), containing 8 experimental groups approximately 32 arrays. The arrays were based on primary human hepatocyte preparations from three liver donors (liner identification numbers: 985, 987, 1002). We chose three groups for the study: the control group consisted of three samples, which were only treated with vehicle (no sulforaphane, SFN) for 48 h; the experimental group was divided into two groups, one group consisted of three samples treated with 10 μM SFN for 48 h, and the other group consisted of three samples treated with 10 μM SFN for 48 h.

### Identification of differentially expressed genes

The differentially expressed genes (DEGs) among three groups (48 h treatment with vehicle only, 48 h treatment with 10 μM SFN and 48 h treatment with 50 μM SFN) were analyzed by ANOVA of R studio with the filtration criteria: adjusted *P*-value <0.05. The DEGs between two groups (48 h treatment with vehicle only and 48 h treatment with 50 μM SFN) were analyzed using Limma package of R studio after normalization and log_2_-transformation of raw microarray data, and the thresholds were |log_2_(fold change [FC])| > 1.5 and adjusted *P*-value <0.05.

### Kyoto Encyclopedia of Genes and Genomes pathway and Gene Ontology enrichment analyses of DEGs

For purpose of evaluating the major functional pathways of primary human hepatocyte after treatment with SFN, the histograms and bubble plots of enriched Gene Ontology (GO) terms and bubble plots were annotated and visualized by the online tool DAVID (https://david.ncifcrf.gov), and Kyoto Encyclopedia of Genes and Genomes (KEGG) pathway and GO annotation analysis of DEGs were assessed via the clusterProfiler and enrichplot package in R studio. *P*<0.05 was considered to be of statistical significance.

### Construction of protein–protein interaction network and hub clusters

The 627 common DEGs, calculated using two different methods (ANOVA and Limma package), were constructed into a protein–protein interaction (PPI) network with the STRING database. Cytoscape software was employed to further analyze the interaction network. The molecular complex detection (MCODE) plugin in cytoscape software was used for the identifition of important molecules in PPI networks under the recognition standard MCODE score ≥ 4 to screen the modules of hub genes. Besides, the drug–gene network was constructed by STITCH to analyze the association between SNF and 64 DEGs in the four hub modules screened by MCODE.

### Prediction of miRNAs targeting HSP90AA1 and construction of miRNA–mRNA network

MiRNAs targeted by HSP90-α (cytosolic), class A member 1 (HSP90AA1) were predicted by miRanda database, and the co-expression network of miRNAs and HSP90AA1 was constructed by R studio. Combined with the Human miRNA Disease Database (HMDD) database, the relationship between HSP90AA1 and miRNAs was further analyzed.

## Results

### Identification of DEGs in 48 h treatment with different concentrations of SFN in human hepatocytes

**Figure 1:**
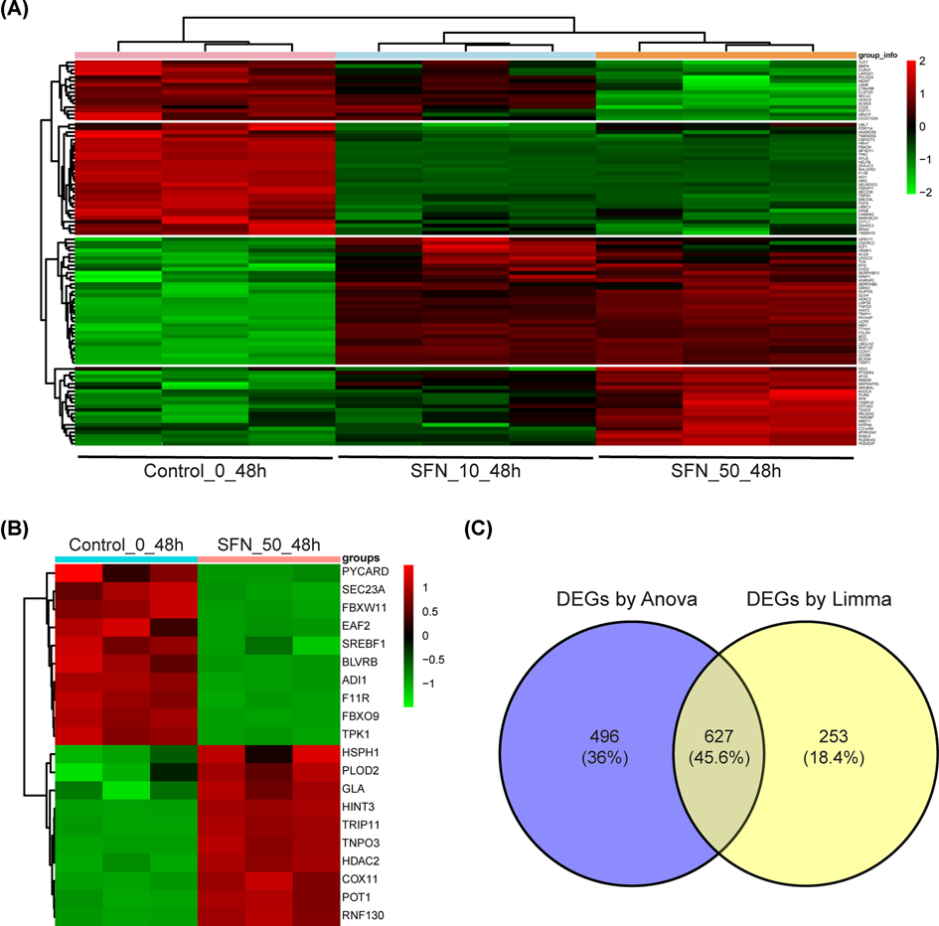
The DEGs in the primary hepatocyte treated with different concentrations SFN were analyzed (**A**) The DEGs in primary hepatocyte cells were showed the top 100 by heat map after SFN treatment with different concentrations. (**B**) The heat map showed the top 20 DEGs in the primary hepatocyte cells treated with 50 μM SFN for 48 h. (**C**) The Venny was used to screen common DEGs calculated using two methods (ANOVA analysis of variance and Limma two-group difference analysis). The adjusted *P*-value was less than 0.05.

For the purpose of evaluating the possible role of SFN in human hepatocytes and elucidating the potential mechanism, the data of microarray GSE20479 in GEO database were analyzed. ANOVA analysis of variance (a 3-group variance test) was used for screening the DEGs among the three group: control group, 48-h treatment with 10 μM SFN and 48-h treatment with 50 μM SFN and the top 100 DEGs were shown by the heat map in the Figure 1A. Besides, Limma package (a 2-group variance test) was used to screen for DEGs in 48 h treatment with 50 μM SFN group compared with the control group. There were 880 DEGs, 501 of which were highly expressed and 379 of which were low-expressed, but only the top 20 genes were shown in the heat map in Figure 1B. The common DEGs, calculated using two different methods (ANOVA and Limma package), were 627 screening by Venny (Figure 1C).

### KEGG pathway enrichment analysis

**Figure 2:**
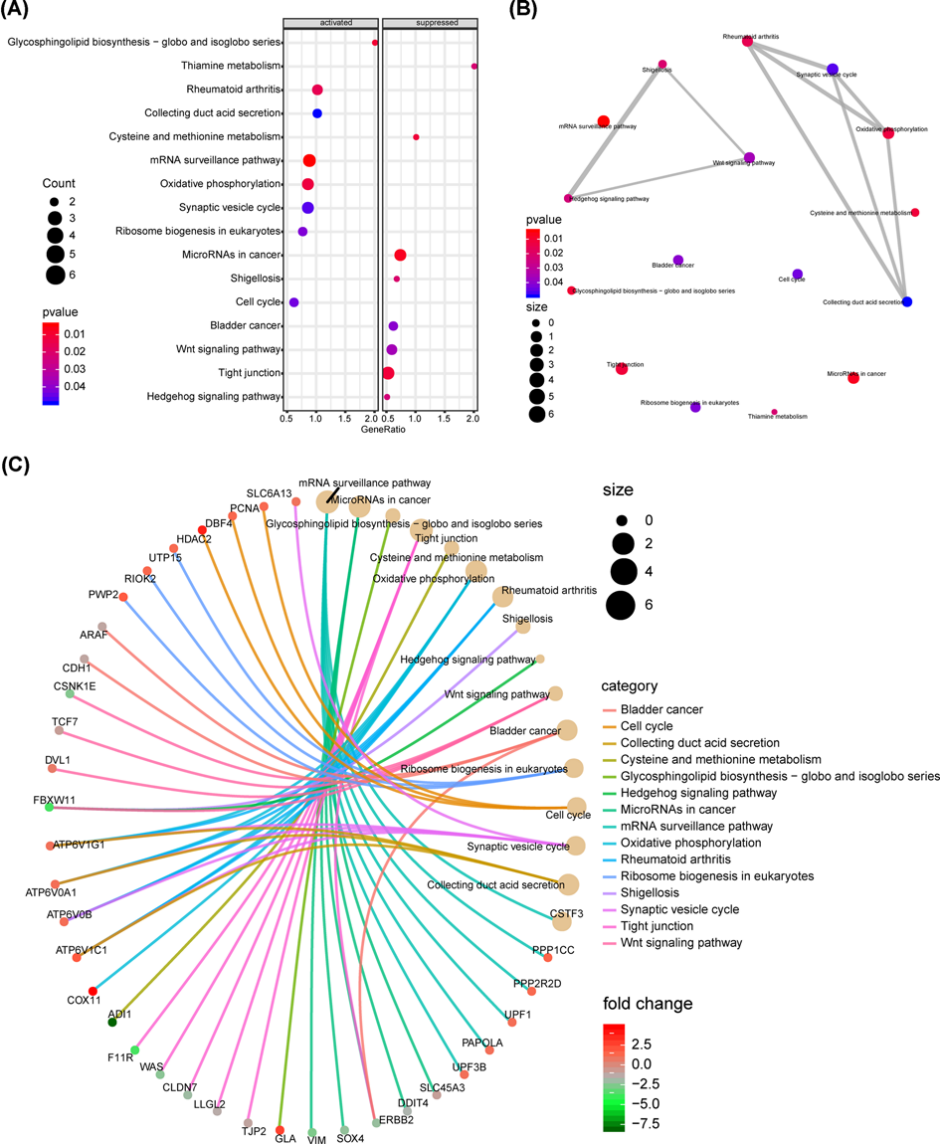
The KEGG pathway enrichment analysis was performed by Clusterprofiler and enrichplot (**A**) The KEGG pathway enrichment analysis of 627 common DEGs was performed by Clusterprofiler and enrichplot, and the results were shown in the dotplot. (**B**) The correlation between enrichment pathways was presented by enrichment map. (**C**) Cnetplot showed the correlation between enrichment pathways and DEGs.

In order to further investigate the function of 627 DEGs, KEGG enrichment analysis was performed to annotate dysfunctional genes and signaling pathways. Clusterprolifer and enrichplot package was employed for KEGG signaling pathway enrichment analysis and the results suggested that microRNAs in cancer pathway was significantly suppressed in 48 h treatment with 50 μM SFN group, while the mRNA surveillance pathway was obviously activated (Figure 2A). The enrichment map showed the correlation among the enrichment pathways. Specifically, WNT signaling pathway, Hedgehog signaling pathway and Shigellosis pathway were associated with each other. Rheumatoid arthritis, Synaptic vesicle cycle, Oxidative phosphorylation and Collecting duct acid secretion were also correlated. This indicated that there was also interaction among signaling pathways (Figure 2B). In addition, the correlation between these enrichment pathways and DEGs was presented by the cnetplot in Figure 2C.

### GO function enrichment analysis

**Figure 3:**
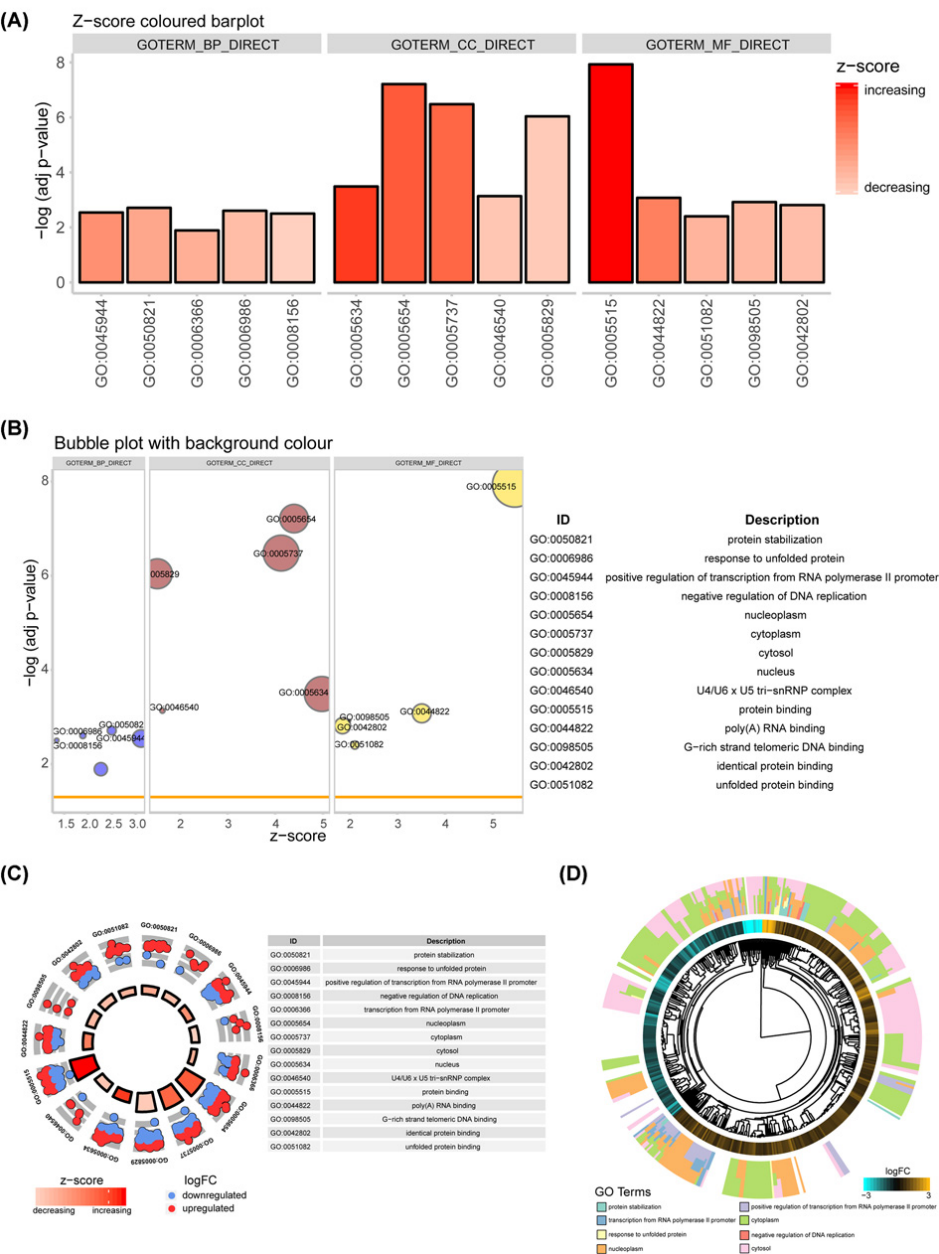
The GO function enrichment analysis was performed by DAVID database and GOplot The results of GO analyses of 627 common DEGs were shown in barplot (**A**) and bubbleplot (**B**), consisting of BP, CC and MF. Circle plot (**C**) and Gocluster plot (**D**) visualized the distribution of high-expression and low-expression genes contained in each term, as well as the Z-score.

GO database was used to describe the role of genes and proteins with three categories information containing biological process (BP), cellular component (CC) and molecular function (MF). DAVID online tool and GOplot visualization package were employed for GO analysis to investigate the function of 627 DEGs. Both of barplot (Figure 3A) and bubble plot (Figure 3B) indicated that DEGs were mainly involved in protein stabilization, response to unfolded protein, positive regulation of transcription from RNA polymerase II promoter and negative regulation of DNA replication in the BP terms. Analysis of CC terms showed that these DEGs mainly concentrated in nucleoplasm, cytoplasm, cytosol and nucleus, which were involved in different MFs such as protein binding, poly(A) RNA binding, G-rich strand telomeric DNA binding, identical protein binding and unfold protein binding. Subsequently, circle plot (Figure 3C) and gocluster plot (Figure 3D) visualized the distribution of up- and down-regulation genes contained in each term, as well as Z-score value. Among them, protein binding (GO: 0005515), cytoplasm (GO: 0005737) and nucleoplasm (GO: 0005654) were the top three prominent terms.

### PPI network analysis of the DEGs and the potential mechanism of HSP90AA1 in 48-h treatment with 50 μM SFN

**Figure 4:**
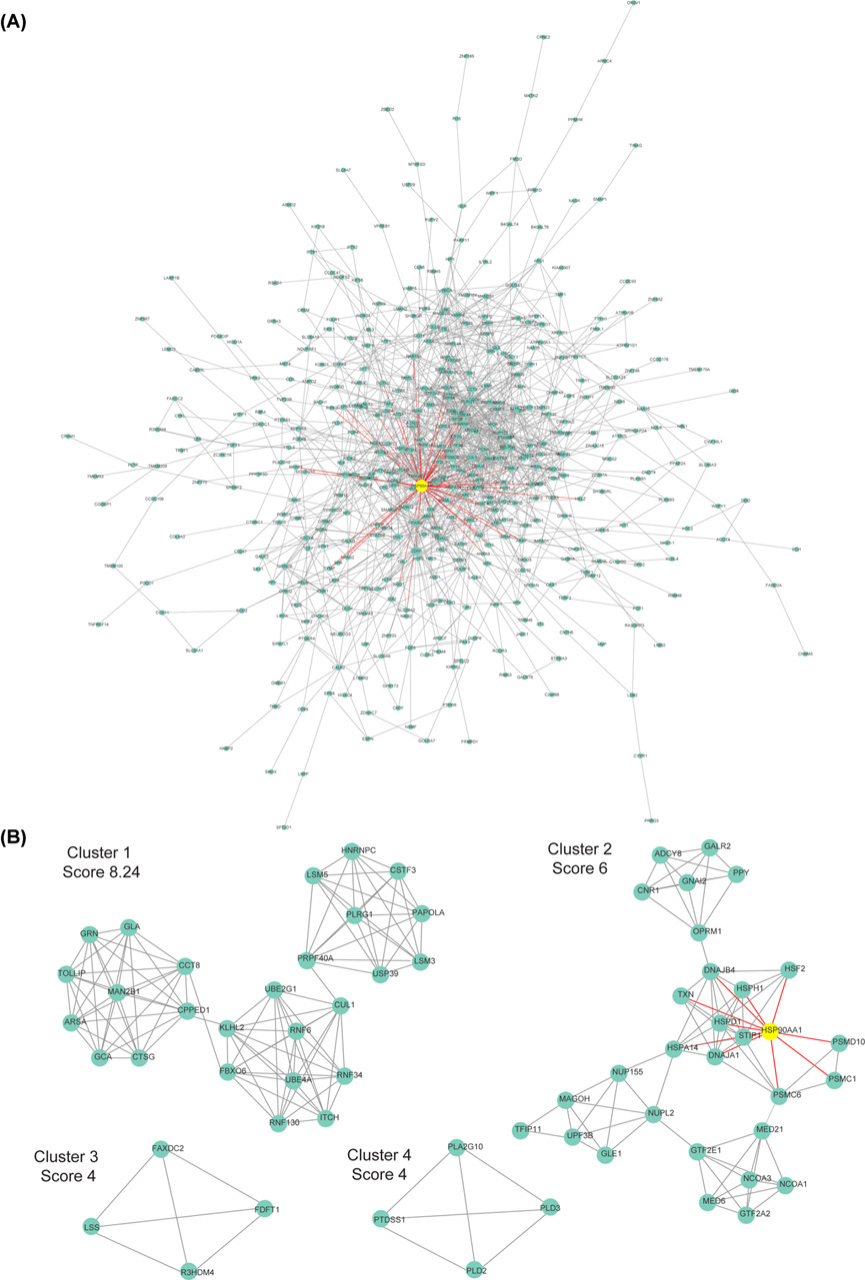
Identification of hub modules from PPI network with the MCODE algorithm (**A**) The PPI network of 627 DEGs was constructed using Cytoscape. (**B**) Four hub modules were extracted from PPI network by using Cytoscape MCODE algorithm. Score > 4.

The PPI network of DEGs was constructed using STRING database, and analyzed by Network analysis in Cytoscape to reveal the potential interaction. HSP90AA1 was found in the central position of the PPI network (Figure 4A). Moreover, MCODE algorithm in Cytoscape was used to identify hub genes in the PPI network. There were four clusters filtrated with Score ≥ 4 as the threshold, in which one of the hub genes was HSP90AA1, an up-regulated gene in 48 h treatment with 50 μM SFN group (Figure 4B).

**Figure 5:**
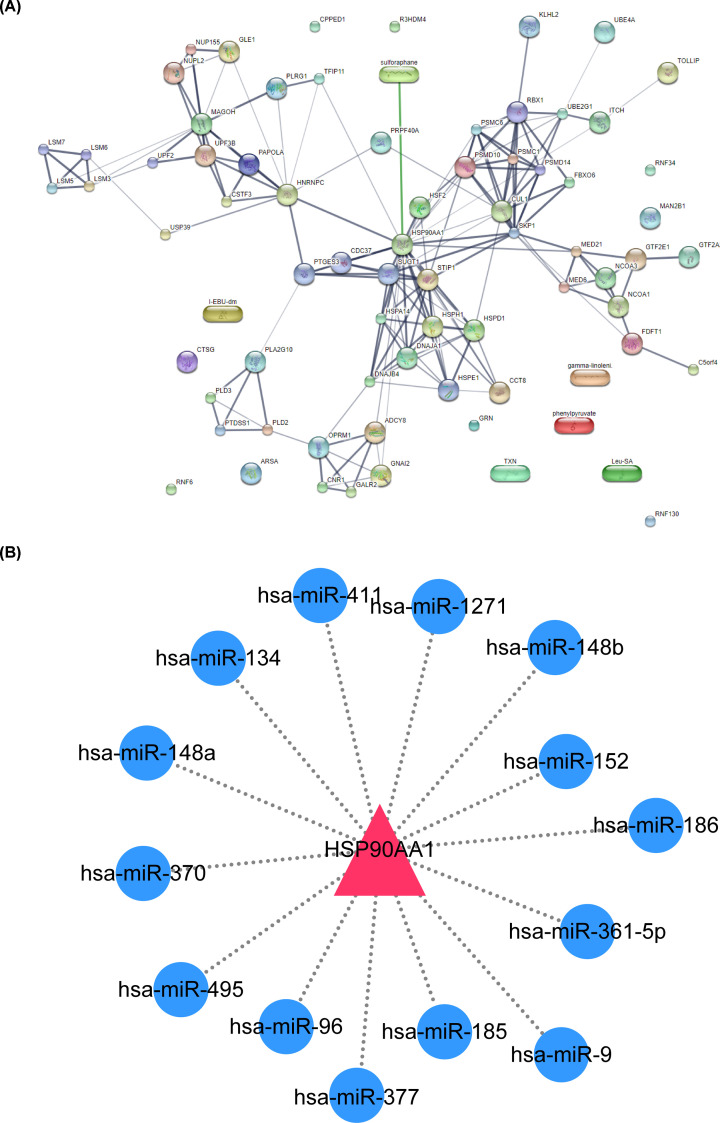
Drug–gene interaction network and potential target miRNAs of SFN were predicted by using STITCH and miRanda database (**A**) The 64 DEGs contained in four hub modules selected by MCODE and SFN constructed the drug–gene network. (**B**) The ceRNA network of HSP90AA1 with HSP90AA1-targeted miRNAs predicted using miRanda database. Circles represent miRNAs and triangles represent HSP90AA1. The nodes highlighted in pink and blue indicate up-regulation and down-regulation, respectively.

The drug–gene network was constructed with the 64 DEGs in four hub clusters filtrated by MOCDE and SFN by STITCH database, which suggested that HSP90AA1 was not only related to SFN but also correlates with many other DEGs, indicating that HSP90AA1 might play a pivotal role (Figure 5A). Based on KEGG pathway enrichment analysis, microRNA in cancer signaling pathway was significantly suppressed in 48 h treatment with 50 μM SFN group. It was speculated that HSP90AA1 might play its role by targeting miRNAs. The miRNAs targeting HSP90AA1 were predicted by using miRanda database and showed in Figure 5B. Furthermore, combined with HMDD database, miR-1271, miR-134 and miR-148a have been proved to have a targeting relationship with HSP90AA1. It indicated that HSP90AA1 might act as a key molecule, through protein interaction and targeted miRNAs, for SFN to function as a tumor suppressor.

## Discussion

With the development of human genome balance and molecular networks, a new concept of disease, network medicine, has emerged, which applies the network concept to the analysis of the dynamic relationship between diseases and drugs, and provides novel opportunities for the development of new treatment methods [18]. SFN is a natural isothiocyanate compound found in cruciferous vegetables, especially broccoli [19]. It has been reported that SFN has been identified as a chemical protective agent with potential clinical application value and beneficial to human health. Moreover, SFN can also inhibit two major CYPs (3A4 and 2D6) in human liver microsomes *in vitro*, namely, the enzymes involved in many important reactions of human drug metabolism [20]. In this study, we studied the changes of gene expression profiles and related pathways or cell functions after SFN treatment of hepatocytes by a series of bioinformatics analysis methods, and preliminarily explore the molecular mechanism of SFN action on hepatocytes.

Hepatocyte culture is a widely accepted method for assessing drug uptake and metabolic mechanism, as well as induction potential of cytochrome P450 *in vitro* [4]. Gross-Steinmeyer et al. found that primary cultures of isolated human hepatocytes were treated with SFN (10 or 50 μM) for 48 h without obvious cytotoxicity observation [14]. Gerhauser et al*.* also indicated that compared with immortalized cell lines such as HepG2 or Hepalc1c7, human hepatocytes seemed to be less sensitive to the cytotoxicity of SFN [21]. In this study, based on the data of GSE20479 chip in GEO database, 627 DEGs in SFN treatment group were screened by ANOVA and Limma. Further KEGG pathway enrichment analysis showed that after 48 h of treatment with 50 mμ SFN, the miRNA signaling pathway was significantly inhibited in hepatocytes, while the RNA signaling pathway was significantly activated. WNT signaling pathway, Hedgehog signaling pathway and Shigellosis pathway are interrelated. There are also correlations among rheumatoid arthritis, synaptic vesicle cycle, oxidative phosphorylation and acid secretion pathways of collecting ducts. In addition, GO functional enrichment analysis showed that 627 DEGs were mainly involved in protein stabilization, unfolded protein response, positive regulation of RNA polymerase II promoter transcription, negative regulation of DNA replication and protein binding. Previous reports have shown that SFN is absorbed by cells, which reduces intracellular glutathione levels, leading to intracellular stress and subsequent activation of various signaling pathways. In addition, SFN has many multi-target effects on cells, including kinase, transcription factor, transporter, receptor, histone deacetylase and tubulin. SFN can also induce autophagy, which is characterized by autophagy formation [15]. Bernkopf et al*.* also pointed out that active WNT/β-catenin signaling pathway is the main driving force of colorectal cancer, while SFN can inhibit the growth of colorectal cancer, although no studies have shown a correlation between the two [12]. Toyama et al*.* found that SFN could inhibit mercury accumulation and toxicity in primary mouse hepatocytes after exposure to MeHg by activating Nrf2 [22]. The strong oxidative effect of SFN has a protective effect on APAP-induced hepatotoxicity [23]. In addition, B6C3F1 mice fed broccoli for a long time could resist the development of NAFLD enhanced by Western diet and liver tumorigenesis induced by DEN [24]. These results and studies suggest that SFN may play a protective role in the formation of hepatocellular carcinoma by regulating certain pathways, thereby affecting the biological function of hepatocytes.

The interaction of 627 DEGs was shown by protein interaction network graph. HSP90AA1, a gene up-regulated in 48 h treatment with 50 μM SFN group, was found in the middle of PPI and was also one of the hub genes. In addition, 64 DEGs from four hub clusters constructed drug–gene networks with SFN. HSP90AA1 was found not only to be the only DEG associated with SFN, but also to be associated with many other DEGs. HSP90AA1, is a subtype of heat shock protein 90 (HSP90) cytosolic, which is encoded on the complement chain of chromosome 14q32.33 and has a full length of more than 59 kbp [25]. It is widely expressed in mammalian cells, but has the highest transcriptional level in brain and testis [26]. As a highly conserved chaperone protein in eukaryotes, HSP90AA1 promotes the maturation of many proteins and plays an important role in many cellular functions [27]. Fang et al*.* reported that HSP90AA1 plays an essential role in a variety of cellular processes including protein folding, protein degradation, signal transduction cascade and morphological evolution, and it is also related to normal protein transport, transcriptional regulation, as well as epigenetic regulation of gene expression [28]. As a new target for cancer therapy, HSP90AA1 is highly expressed in a variety of malignant tumors, including breast cancer, endometrial cancer, ovarian cancer, colon cancer, lung cancer and prostate cancer [29]. For example, Berglund et al*.* demonstrated that HSP90AA1 was up-regulated during chemotherapy in osteosarcoma patients, which promoted autophagy and inhibited apoptosis through PI3K/Akt/mTOR pathway and JNK/p38 pathway respectively, so as to promote the chemotherapeutic resistance of osteosarcoma both *in vivo* and *in vitro* [30]. These results suggested that HSP90AA1 might be down-regulated by SFN in hepatocytes. However, Hahm et al*.* reported the contradict conclusion that the protein level of HSP90AA1 in PC-3 cells increased after SFN treatment for 6 h [31], which was consistent with our analysis results.

Finally, in order to further explore the regulatory mechanism of HSP90A11, we preliminarily screened out the targeted HSP90AA1 and HCC-related miRNAs based on HMDD database and miRanda database. Moreover, the expression of HSP90AA1 has been proved to be regulated by miR-10a and miR-204 that are significantly correlated with the radiosensitivity and survival rate of patients with low-grade neoplasia, which can be used as a clinical biomarker for prognosis and diagnosis of low-grade neoplasia [32]. In addition, Dong et al*.* found that HSP90AA1 expression was regulated by miR-27b-3p sponge KCNQ1OT1, and affected the migration and invasion ability of NSCLC cells [33].

In conclusion, HSP90AA1 may be a key molecule of SFN in the development of hepatocellular carcinoma, which plays a role through protein interaction and subsequent changes in signal pathway status. However, the specific mechanism of SFN acting through HSP90AA1 still needs to be further validated at the cellular and animal levels.

## Data Availability

All data were presented in the article. GSE20479 based on the platforms—GPL1313 was obtained from GEO public database (https://www.ncbi.nlm.nih.gov/geo/), containing 8 experimental groups in approximately 32 arrays.

## Abbreviations

APAP: acetaminophen
BBB: blood-brain barrier
BP: biological process
CC: cellular component
CSC: cancer stem cell
DEG: differentially expressed gene
GEO: Gene Expression Omnibus
GLS: glucosinolates
GO: gene ontology
HCC: hepatocellular carcinoma
HMDD: Human miRNA Disease Database
HSP90: heat shock protein 90
HSP90AA1: HSP90-α (cytosolic), class A member 1
KEGG: Kyoto Encyclopedia of Genes and Genomes
MCODE: molecular complex detection
MF: molecular function
Nrf2: nuclear factor-erythroid 2-related factor 2
NSCLC: non-small cell lung cancer
PPI: protein–protein interaction
SFN: sulforaphane; 4-methylsulfinylbutyl isothiocyanate
SFN-Cys: SFN-cysteine
SFN-NAC: SFN-N-actyl-cysteine
STRING: Search Tool for the Tetrieval of Interacting Genes
WNT: Wingless/Integrated
